# Effects of rear-foot instability devices on lower-limb muscle activation during the Bulgarian split squat in male football players

**DOI:** 10.1038/s41598-025-32203-7

**Published:** 2025-12-14

**Authors:** Hüseyin Topçu, Ali Kamil Güngör, Yahya Yıldırım, Ufuk Şekir, David G. Behm, Monira I. Aldhahi

**Affiliations:** 1 Department of Physical Education and Sport, Faculty of Sport Sciences, Bursa Uludag University, Bursa, Turkey; 2 Department of Coaching Education, Faculty of Sport Sciences, Bursa Uludag University, Bursa, Turkey; 3 Department of Sports Medicine, Faculty of Medicine, Bursa Uludag University, Bursa, Turkey; 4 School of Human Kinetics and Recreation, Memorial University of Newfoundland, St. John’s, Newfoundland and Labrador, St. John’s, Canada; 5 Department of Rehabilitation Sciences, College of Health and Rehabilitation Sciences, Princess Nourah bint Abdulrahman University, P.O. Box 84428, Riyadh, 11671 Saudi Arabia

**Keywords:** EMG, Unstable surface, BOSU ball, Resistance training, Swiss ball, Anatomy, Health care, Physiology

## Abstract

Unilateral resistance exercises such as the Bulgarian Split Squat (BSS) are commonly used to develop lower-limb strength, postural control, and neuromuscular coordination, depending on training variables (e.g., load and intensity). Although instability training increases muscle activation, few studies have examined the effect of rearfoot instability on neuromuscular responses during BSS. This randomized crossover study investigated the acute effects of three rear-foot instability devices on muscle activation during the ascent and descent phases of the BSS in 23 trained male football players. Participants performed body-weight BSS under four conditions: stable platform, BOSU ball, Swiss ball (Swiss), and TRX suspension. Surface electromyography (sEMG) recorded activation of the rectus femoris (RF), vastus lateralis (VL), vastus medialis (VM), biceps femoris (BF), semitendinosus (ST), and gluteus maximus (GM). Two‑way repeated‑measures ANOVA showed significantly greater activation during ascent for BF (*p* < 0.001), ST (*p* = 0.006), VL (*p* < 0.001), VM (*p* < 0.001), and GM (*p* < 0.001). Quadriceps activation during descent was highest on the Swiss: RF (Swiss vs. stable: *p* = 0.002; Swiss vs. BOSU: *p* < 0.001; Swiss vs. TRX: *p* = 0.006), VL (Swiss vs. stable: *p* = 0.017; Swiss vs. BOSU: *p* = 0.001), and VM (Swiss vs. stable: *p* = 0.024; Swiss vs. BOSU: *p* = 0.046). TRX increased ST activation during the ascent compared to the Swiss (*p* = 0.034), and the BOSU showed higher ST activation than the Swiss during the descent (*p* = 0.004). Surface significantly affected activation (ST: *p* = 0.018; RF: *p* < 0.001; VL: *p* < 0.001; VM: *p* = 0.013; GM: *p* = 0.042), and there was a significant surface × phase interaction for GM (*p* = 0.041). The findings highlight rearfoot instability as an effective programming variable to selectively enhance muscle activation without external loading, supporting its application in strength and rehabilitation programs.

## Introduction

Resistance training plays a fundamental role in athletic development by improving physical performance and contributing to injury prevention in football^1,2^. However, traditional resistance exercises may not fully address the complex and multidirectional demands of sport-specific tasks^3^, highlighting the need for more transferable training strategies to improve performance. In response, contemporary strength and conditioning practices have increasingly emphasized the integration of functional resistance training modalities^4,5^. These approaches have been effective in enhancing sport-specific motor abilities, such as acceleration, deceleration, change of direction, and vertical jump performance^6,7^. Such movements are frequently observed in competitive sports and are critical for success. These high-demand tasks often require unilateral loading patterns and single-limb force production. Compared with traditional bilateral exercises, such training more accurately replicates the biomechanical and neuromuscular demands of many sport-specific actions^8,9^.

The Bulgarian Split Squat (BSS) is widely employed in unilateral resistance training and is recognized for its potential to enhance unilateral strength, correct inter-limb asymmetries, and improve dynamic stability, while acknowledging that these adaptations are also influenced by other factors (e.g., training load, duration, intensity, and individual variability)^8,10,11^. The BSS involves positioning the rear foot on an elevated surface while the lead leg performs a squat movement with either body mass or an additional load as resistance. The BSS can be performed with variations in rearfoot elevation, trunk angle, or the use of additional load. The BSS with its more limited base of support and sport specificity (i.e., unilateral movement) can challenge balance and neuromuscular control (i.e., stability, coordination)^12^. Although inherently less stable than bilateral exercises, the BSS supports sport-specific tasks such as jumping and change of direction and may reduce inter-limb imbalances^13^. Some studies have reported greater muscle activation in key lower limb muscles, such as the gluteus, quadriceps and hamstrings, during the BSS than during bilateral exercises^12,14^. However, other studies have reported no significant differences or even lower activation levels under similar conditions^15^, indicating inconsistencies in the literature regarding the neuromuscular demands of BSS.

In response to the growing interest in enhancing neuromuscular coordination and balance control (exercises that stress vestibular and proprioceptive responses), practitioners have increasingly integrated instability-based tools such as BOSU balls, Swiss balls, and suspension training systems like TRX into unilateral exercise protocols^6,16,17^. It has been hypothesized that performing exercises under unstable conditions facilitates greater motor unit recruitment, particularly in stabilizing musculature^18–21^. However, the evidence supporting this claim remains mixed and appears to be highly context-dependent^16^. For example, McBride et al.^22^ reported significantly greater activation of the biceps femoris (BF) during squats performed on dual inflatable balance discs in recreationally trained males. In contrast, Saeterbakken and Fimland^23^ found no significant differences in the activation of the vastus lateralis (VL) and vastus medialis (VM) when squats were performed on various unstable surfaces, including the Power Board, BOSU, and Balance Cone. These contrasting findings suggest that the neuromuscular response to instability may be muscle-specific and influenced by the nature and degree of instability. Furthermore, the trained state of the individual may also play a role as resistance trained individuals did not experience increased muscle activation when performing resistance exercises (e.g., squats) on moderately unstable surfaces^24^.

Although instability training has received growing interest in recent years, research specifically examining its effects on BSS remains limited and inconsistent. Notably, most existing studies have focused on anterior (lead foot) instability, whereas the influence of rear-foot instability on neuromuscular responses has not been extensively explored^6,25^. This constitutes a notable gap in the literature, especially given the critical role of the rearfoot in maintaining postural stability and regulating load distribution throughout the BSS. There remains a lack of consensus regarding whether instability positively influences or impairs neuromuscular activation during the eccentric (descending) and concentric (ascending) phases of movement. Furthermore, force output exerted while unstable can be severely diminished (e.g., 60% decrease with chest press on Swiss ball)^16,18–21,26–28^ and thus exercises that can improve balance and stability would increase force output (strength). To the best of our knowledge, this is the first study to comprehensively investigate phase-specific neuromuscular responses to three distinct rearfoot instability devices during the BSS.

Therefore, the primary aim of this study was to examine the effects of different rear-foot instability devices on lower extremity muscle activation during BSS. Additionally, we aimed to compare muscle activation levels between the ascending and descending phases of the BSS under stable and unstable conditions. We hypothesized that unstable devices would elicit greater muscle activation than stable surfaces and that the ascending phase would demonstrate higher muscle activation than the descending phase, regardless of the surface condition.

## Methods

### Study design

A randomized, repeated-measures crossover design was employed to examine lower limb muscle activation during the BSS performed with the rear leg on four surfaces: a stable platform, a BOSU ball, a Swiss ball, and TRX suspension straps. To mitigate potential carryover and fatigue effects, four predetermined balanced orders (based on a Latin square) were prepared. Participants randomly selected a card-draw method and assigned themselves to one of these balanced orders. Each participant completed all four conditions in a single testing session with standardized rest intervals (2 min) between trials^29^. All testing sessions were conducted between 10:00 a.m. and 12:00 p.m. to control for the effects of circadian rhythm on neuromuscular activation. The subsequent sections detail the testing procedures, electrode placement, signal-processing techniques, and statistical analyses. Written informed consent was obtained from all participants in accordance with the Declaration of Helsinki, and the study was approved by the Local Clinical Research Ethics Committee (Decision Number: 2022-09/12).

### Participants and sample size calculation

A priori power analysis was conducted using G Power software (version 3.1.9.7) for the F-test family (repeated measures ANOVA, within‐subject factors) to determine the required sample size. Assuming a medium effect size f = 0.25, α = 0.05, power of β = 0.80, number of groups = 1, number of measurements = 4, correlation among repetition measures = 0.5, and nonsphericity correction ε = 1, the required sample size was calculated as 24. In accordance with the sample size estimation suggested by the G*Power analysis, the study initially included 24 participants; however, owing to the withdrawal of one participant during the experimental phase, the final analyses were conducted with data from 23 male participants (mean age = 22.7 ± 2.34 years, height = 180.6 ± 3.85 cm, body mass = 76.45 ± 3.90 kg, and BMI = 23.43 ± 1.49 kg/m²). The inclusion criteria for the participants were as follows: (a) at least three years of sports background (soccer), (b) classified as sub-elite players who trained at least four times per week and played one official match weekly, and (c) experience with the BSS. The exclusion criteria were as follows: (a) joint or bone injury in the last six months, (b) musculoskeletal problems (pain, injuries) that might influence performance, and (c) use of drugs or similar substances (stimulants) that affect the musculoskeletal system. To ensure consistent performance, the participants were instructed to avoid strenuous physical activity and alcohol consumption for at least 24 h before each testing session.

Additionally, they were advised to refrain from using tobacco and consuming caffeine-containing products for at least three hours before each session. One week before the experimental session, the participants completed a familiarization session to practice the maximal voluntary isometric contraction (MVIC) and BSS exercises on each of the four surfaces. The purpose of this session was to ensure proper technique and familiarize the participants with the testing procedures. The actual MVIC tests used for the normalization of surface electromyography (sEMG) signals were conducted on the day of the experimental session prior to the exercise trials.

### Procedures

The research consisted of two sessions, namely the familiarization and experimental sessions, which took place between 10:00 a.m. and 12:00 p.m. and were separated by one week. During the familiarization session, various measurements were taken, including age, weight, height, and leg length, which was defined as the distance between the anterior superior iliac spine and the medial malleolus of the tibia. Participants were asked which leg they would use to kick a ball to determine leg dominance, and the dominant leg was positioned as the front (loading) leg to ensure methodological consistency and to minimize inter-limb variability. The dominant limb generally provides greater motor control and stability during unilateral loading, which helps reduce postural adjustments and improves the reliability of EMG measurements^30–32^. To ensure adherence to the pre-test instructions, the participants completed a standardized questionnaire. All participants performed the exercises wearing their own athletic shoes, which were typical training footwear that provided standard grip and stability. None of the participants wore braces, compression garments, or other accessories that could have influenced the sEMG recordings. To familiarize the participants with the exercise procedures, they performed two sets of six repetitions of BSS exercises on four surfaces (Stable, Bosu, Swiss ball, and TRX) to achieve the proper technique before data collection. In addition, the methods to be applied in the MVIC measurement procedure were tested during the familiarization sessions.

During the experimental trial, surface electrodes were attached for the MVIC test. Before the MVIC test, the participants performed a 10-minute warm-up on a treadmill at an intensity corresponding to a rating of perceived exertion between 8 and 10 on the Borg scale (from no effort 6 to maximum effort 20)^33,34^. The Borg scale was as follows: extremely light (7–8), very light (9–10), light (11–12), somewhat hard (13–14), hard (15–16), very hard (17–18), and extremely hard (19). Following the MVIC test protocol, the participants were instructed to perform three consecutive BSS repetitions. Each repetition comprised a 2-second descent and a 2-second ascent. The KORG MA-1 metronome provided auditory cues to standardize the timing of both phases, ensuring that the participants initiated the descent and ascent at the correct intervals for each repetition. Trials in which the participants could not execute the exercises using the appropriate technique and pace were excluded and repeated. The participants were familiar with the exercises; however, for safety reasons, they were reminded of three key points: maintaining heel contact with the floor, keeping the knees aligned with the toes, and preserving the natural lumbar curvature. If any of these points were violated, the test was terminated. The primary objective was to complete different tasks at a controlled pace while maintaining a consistent posture. The BSS was performed under four conditions: rear leg on a stable surface, BOSU, Swiss ball, and TRX suspension trainer.

The ascent and descent phases were determined by visually analyzing the video recordings of the participants. The lowest point of the movement was defined as the transition between the descent and ascent phases. EMG signals and video data were recorded simultaneously using the same system (ME6000; Mega Electronics Ltd., Kuopio, Finland) and software (MegaWin) that provides automatic temporal synchronization via integrated timestamping. Therefore, no additional hardware triggers were required. The timing alignment error between the EMG and video data was below one frame (± 0.03 s).

**Fig. 1:**
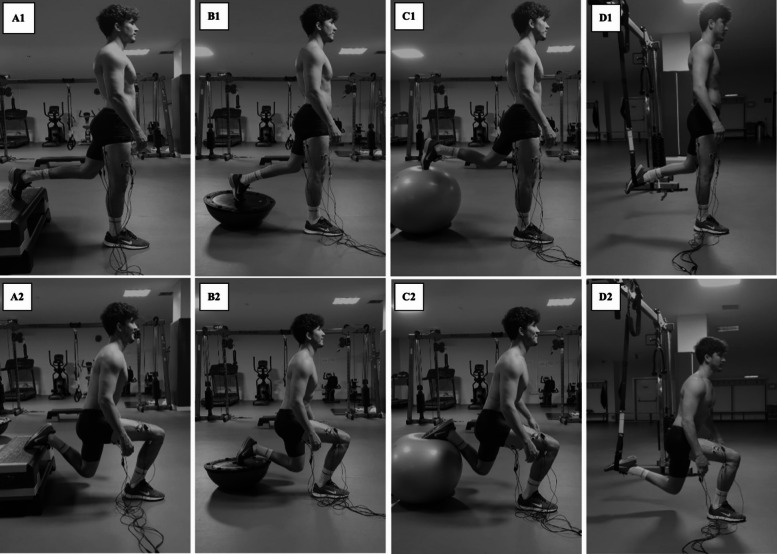
Bulgarian Split Squat on four rear-foot support surfaces. **A1-2** = Stable; **B1-2** = BOSU; **C1-2** = Swiss Ball; **D1-2** = TRX (Picture used with permission).

*Exercise Description.* The surfaces were adjusted to 60% of the participants’ leg length to standardize the height and stepping distance for all BSS conditions. In comparison, the stepped distance was set at 80% of the participant’s leg length^35^, as measured from the anterior superior iliac spine to the medial malleolus of the tibia by Boudreau et al.^36^. BSS exercise was performed under all conditions using the participants’ body weight. No additional weight was used. The participants were directed to assume an upright stance with one foot positioned anteriorly and the other posteriorly. The participants maintained a proper stance with their arms hanging at their sides and natural lumbar lordosis throughout the exercise. The participants performed the Bulgarian Split Squat in a vertical (up–down) manner, maintaining an upright trunk, with the rear leg placed on the instability device. During the descent phase, the participants lowered their bodies until the knee of the forward leg flexed to 90˚. In the subsequent ascent phase, the participants returned to the initial position with complete extension of the forward leg’s knee while maintaining an erect trunk posture, as per the guidelines provided. The front foot was positioned flat on the floor during each trial and remained fixed throughout the trial. In contrast, the rear foot was positioned with the toes pointing downward so that the forefoot contacted one of the four instability devices (step board, BOSU ball, Swiss ball, or TRX) assigned in a randomized order. In the TRX condition, the foot was placed in a suspension strap with the toes facing downward (Fig. 1). The height of the rear leg was adjusted using a stepboard. The contact point between the devices and the feet was standardized for all repetitions.

*Surface Electromyography.* All sEMG values were recorded using a portable 8-channel surface sEMG device (ME6000; Mega Electronics, Kuopio, Finland) at a sampling frequency of 1000 Hz. Data were analyzed using the MegaWin software (Mega Electronics Ltd.). The sEMG signals were bandpass-filtered at 20–500 Hz using a 4th-order Butterworth filter^37^. The root mean square (RMS) of the sEMG signals was calculated for the duration of each exercise. The mean RMS values were then normalized to each participant’s MVIC and expressed as a percentage of MVIC (%MVIC).

This study used bipolar sEMG electrodes with an inter-electrode distance of 2 cm. These surface electrodes were placed on the BF, Semitendinosus (ST), Rectus Femoris (RF), VL, VM, and Gluteus Maximus (GM) muscles of the dominant leg. Prior to electrode placement, the participant’s skin sites were prepared by shaving and cleaning with alcohol to reduce the impedance caused by dead surface tissue and oil. The order of electrodes followed the recommendations of the SENIAM project^38^. The RF electrode was positioned at 50% of the line from the anterior superior iliac spine (ASIS) to the superior border of the patella. The VL electrode was placed at two-thirds of the line from the ASIS to the lateral patella, and the VM electrode was placed at 80% of the line from the ASIS to the joint space anterior to the medial ligament. For the BF, the electrode was positioned at 50% of the line between the ischial tuberosity and the lateral tibial epicondyle, whereas the ST electrode was placed at 50% of the line from the ischial tuberosity to the medial tibial epicondyle. The GM electrode was positioned midway between the sacrum and the greater trochanter, over the thickest portion of the muscle belly and aligned with the muscle fibers. Light isometric contractions were performed to confirm that the electrodes activated the intended muscle, and the EMG signal patterns were visually inspected to verify minimal crosstalk.

During the MVIC protocol, the participants performed two separate 5-second MVICs for each muscle group, and the attempt that yielded the greater RMS EMG signal was chosen for analysis. As recommended by previous studies, the RMS EMG signals were normalized to the highest 3-second (s) of the participants’ 5-second MVICs^14,38,39^. The participants were instructed to gradually increase the force of muscle contraction to a maximum over 2 s, sustain the MVIC for 3 s, and slowly release the force. A rest period of one minute was provided between each MVIC^25^, and standardized verbal encouragement was given to motivate the participants to achieve maximal muscle activation. The positions used during the MVICs were based on the Konrad for the dominant leg muscles, including the BF, ST, RF, VL, VM, and GM muscles^40^. To obtain the MVIC for the knee extensor muscles, the participants performed an isometric 90-degree single-leg knee extension in a seated position against matched resistance. The resistance was matched using an ankle cuff attached to a cable anchored to a bolt to ensure a fixed position. For the knee flexor muscles, the participants performed an isometric 20–30 degree single-knee flexion in a prone-lying position against matched resistance. To test the GM, the participants were positioned prone with the testing limb at approximately 0 °of hip extension, and isometric hip extension was performed against matched resistance. Exercise trials were performed after all the MVICs were collected. In this study, the RMS EMG signal of each muscle was obtained during three repetitions of the BSS, and the trial with the higher RMS signal was selected for further analysis^23^.

### Statistical analysis

Descriptive parameters are presented as means and standard deviations, and the normality of the data was assessed using the Shapiro-Wilk test. A 2-way analysis of variance (ANOVA) with repeated measures (4 surfaces × 2 phases) was used to evaluate the differences in EMG activity for each muscle during the exercises. Mauchly’s test was used to assess sphericity for the surface factor and surface × phase interaction; Greenhouse–Geisser corrections were applied when violated. The independent variables were phase (descent and ascent) and surface (stable, BOSU, Swiss Ball, and TRX). The significance criterion was set at *p* < 0.05. Bonferroni post hoc tests were employed to identify significant differences. To test the effect sizes of repeated measures’ main effects and interactions, partial eta squared ($\:{\eta\:}_{p}^{2}$) was used, with values of 0.01, 0.06, and 0.14 indicating small, medium, and large effects, respectively^41^. The data were reported as mean ± 95% confidence intervals (CI) and with Cohen’s d effect size (ES), which was evaluated based on the following criteria: an ES of < 0.2 was considered trivial, 0.2 and < 0.5 was considered small, 0.5 and < 0.8 was considered medium, and > 0.8 was considered large^42^.

A *post hoc* power analysis was performed using G Power software (version 3.1.9) to determine the statistical power (1 − β) of the repeated measures ANOVA with four measurements for a sample size of 23. The analysis assumed a correlation among repeated measures of 0.5 and a medium effect size (*f* = 0.25), and the resulting statistical power was 0.81.

## Results

The main effects and interactions of the phases and surfaces of all the muscles are presented below. The pairwise comparisons are listed in Table 1.

**Table 1:** The muscle activity (% of MVIC) with a 95% confidence interval in the descent and ascent phase of the BSS exercise on different surfaces.

Muscles	Phases	Surfaces								
		Stable (1)	BOSU (2)	Swiss Ball (3)	TRX (4)					
		**Mean (SD)**	**Mean (SD)**	**Mean (SD)**	**Mean (SD)**	**Difference**	**∆% (**↑↓**)**	**(95% CI)**	**p value**	**Cohen’s** ***d*** **(95% CI)**
BF	Descent **(a)**	8.41 (3.80)	9.82 (5.78)	9.77 (4.86)	8.95 (4.29)	**a1 - b1** **a2 - b2** **a3 - b3** **a4 - b4**	29.72 ↑ 34.72 ↑ 27.94 ↑ 34.07 ↑	(-3.796 to -1.204) (-4.754 to -2.064) (-3.922 to -1.533) (-4.403 to -1.688)	0.004 0.001 0.001 0.001	-0.502 ( -1.029 to 0.024) -0.685 (-1.284 to -0.086) -0.548 (-1.060 to -0.037) -0.612 (-1.190 to -0.034)
	Ascent **(b)**	10.91 (4.32)	13.23 (6.09)	12.50 (5.57)	12.00 (4.63)					
ST	Descent **(a)**	12.45 (7.39)	12.86 (6.83)	10.18 (5.00)	11.64 (5.66)	**a2 - b2** **a3 - b3** **a4 - b4** **a2 – a3** **b3 – b4**	25.50 ↑ 25.04 ↑ 39.00 ↑ 20.83 ↓ 27.10 ↑	(-5.643 to -0.902) (-4.307 to -0.784) (-6.494 to -2.597) (0.724 to 4.639) (-6.716 to -0.193)	0.009 0.007 < 0.001 0.004 0.034	-0.447 (-1.030 to 0.135) 0.348 (-0.912 to 0.216) -0.621 (-1.245 to 0.002) 0.367 (-0.176 to 0.909) -0.472 (-1.036 to 0.092)
	Ascent **(b)**	13.95 (8.61)	16.14 (9.48)	12.73 (6.09)	16.18 (8.30)					
RF	Descent **(a)**	17.68 (10.01)	18.18 (9.47)	26.00 (10.12)	19.55 (10.99)	**a1 – b1** **a4 – b4** **a1 – a3** **a2 – a3** **a3 – a4** **b2 – b3**	24.43 ↑ 16.47 ↑ 47.05 ↑ 43.01 ↑ 24.80 ↓ 29.75 ↑	(-8.355 to -0.281) (-5.613 to -0.842) (-13.856 to -2.780) (-11.939 to -3.697) (1.533 to 11.376) (-10.479 to -1.339)	0.037 0.010 0.002 < 0.001 0.006 0.007	-0.406 (-1.095 to 0.284) -0.303 (-0.723 to 0.117) -0.781 (-1.552 to -0.011) -0.734 (-1359 to -0.110) 0.606 (-0.052 to 1.265) -0.555 (-1.165 to 0.054)
	Ascent **(b)**	22.00 (11.11)	19.86 (10.52)	25.77 (10.89)	22.77 (11.86)					
VL	Descent **(a)**	43.64 (25.58)	45.45 (21.58)	53.36 (23.02)	47.55 (21.71)	**a1 - b1** **a2 - b2** **a3 - b3** **a4 - b4** **a1 – a3** **a2 – a3**	31.87 ↑ 32.60 ↑ 20.78 ↑ 31.92 ↑ 22.27 ↑ 17.40 ↑	(-20.713 to -7.105) (-21.487 to -8.149) (-15.723 to -6.459) (-21.413 to -8.951) (-18.128 to -1.327) (-12.982 to -2.837)	< 0.001 < 0.001 < 0.001 < 0.001 0.017 0.001	-0.532 (-1.066 to 0.003) -0.566 (-1.104 to -0.028) -0.424 (-0.808 to -0.040) -0.580 (-1.100 to -0.061) -0.372 (-0.816 to 0.073) -0.302 (-0.593 to -0.012)
	Ascent **(b)**	57.55 (23.94)	60.27 (31.67)	64.45 (28.69)	62.73 (30.94)					
VM	Descent **(a)**	47.91 (23.06)	52.00 (24.76)	63.59 (34.90)	55.82 (30.86)	**a1 - b1** **a2 - b2** **a3 - b3** **a4 - b4** **a1 – a3** **a2 – a3**	44.02 ↑ 31,28 ↑ 17,58 ↑ 37,45 ↑ 32,72 ↑ 22,28 ↑	(-30,165 to -12,017) (-26.388 to -6.157) (-17.486 to -4.878) (-34.268 to -7.550) (-31.183 to -0.181) (-22.048 to -1.134)	< 0.001 0,003 0.001 0,004 0,046 0,024	-0.614 (-1.180 to -0.047) -0.473 (-1.043 to 0.096) -0.325 (-0.688 to 0.037) -0.608 (-1.356 to 0.139) -0.456 (-1.064 to 0.152) -0.337 (-0.754 to 0.080)
	Ascent **(b)**	69.00 (32.05)	68.27 (37.17)	74.77 (37.57)	76.73 (48.15)					
GM	Descent **(a)**	11.59 (5.43)	14.27 (8.51)	11.00 (5.04)	10.41 (4.23)	**a1 - b1** **a2 - b2** **a3 - b3** **a4 - b4**	129,42 ↑ 98,73 ↑ 180,18 ↑ 182,90 ↑	(-19.184 to -10.816) (-19.497 to -8.685) (-24.880 to -14.756) (-22.946 to -15.145)	< 0.001 < 0.001 < 0.001 < 0.001	-1.456 (-2.520 to -0.392) -1.367 (-2.543 to -0.192) -1.923 (-3.279 to -0.567) -1.848 (-3.058 to -0.639)
	Ascent **(b)**	26.59 (12.04)	28.36 (13.29)	30.82 (15.60)	29.45 (11.81)					

BF: Biceps Femoris, ST: Semitendinosus, RF: Rectus Femoris, VL: Vastus Lateralis, VM: Vastus Medialis, GM: Gluteus Maximus, CI: Confidence Interval, SD: Standard Deviation.

### Biceps femoris

**Fig. 2:**
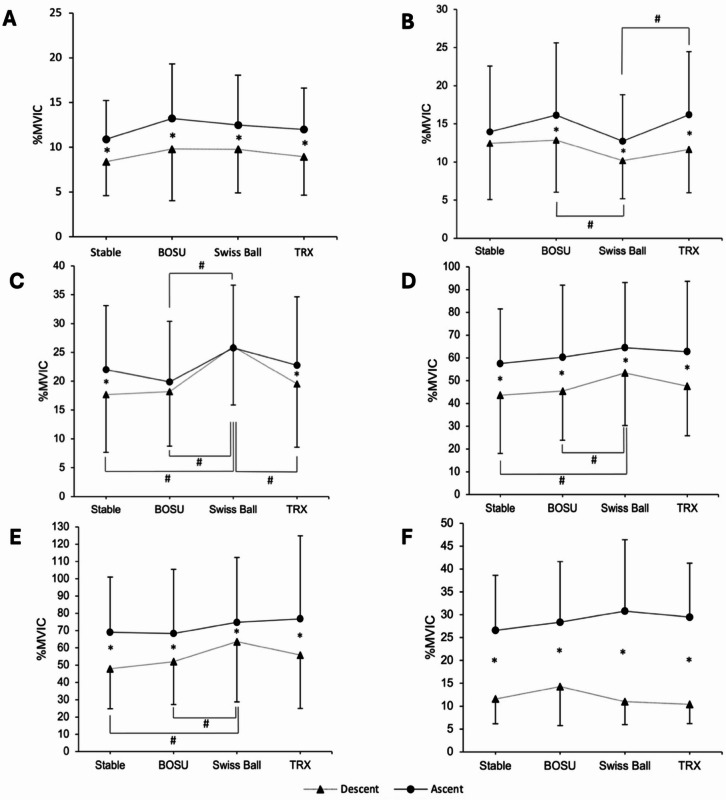
Normalized EMG activity (%MVIC) of six lower-limb muscles during the Bulgarian Split Squat across four rear-foot support surfaces. Muscles: A = Biceps Femoris, B = Semitendinosus, C = Rectus Femoris, D = Vastus Lateralis, E = Vastus Medialis, F = Gluteus Maximus. * *p* < 0.05 between phases; # *p* < 0.05 between surfaces.

No significant main effects of the surface [*F*_(3,63)_ = 2.435, *p* = 0.07] were observed. The results indicate significant main effects of the phases [*F*_(1,21)_ = 48.172, *p* < 0.001, $\:{\eta\:}_{p}^{2}$ = 0.696], with greater EMG activity observed in the BF during the ascent phase compared to the descent phase (*p* < 0.05) (Fig. 2-A). No significant interaction was observed between the surfaces and phases [*F*_(3, 63)_ = 0.548, *p* = 0.651, $\:{\eta\:}_{p}^{2}$*=* 0.025].

### Semitendinosus

Significant main effects of surfaces [*F*_(3,63)_ = 3.608, *p* = 0.018, $\:{\eta\:}_{p}^{2}$ = 0.147] indicated that the BOSU ball demonstrated higher ST EMG activity than the Swiss ball condition (*p* < 0.05). Significant main effects of phases [*F*_(1,21)_ = 9.163, *p* = 0.006, $\:{\eta\:}_{p}^{2}$ = 0.304] were observed, indicating greater EMG activity in the ST muscle during the ascent phase compared to the descent phase (*p* < 0.05). No significant interaction between surfaces and phases [*F*_(1.97, 41.44)_ = 3.025, *p* = 0.060, $\:{\eta\:}_{p}^{2}$ = 0.126] was observed. Pairwise comparisons showed that participants exhibited greater EMG activation during the descent phase on the BOSU ball than on the Swiss ball (*p* < 0.05) and during the ascent phase on the TRX than on the Swiss ball. Additionally, the ascent phase elicited higher EMG activation than the descent phase on the BOSU ball, Swiss ball, and TRX (*p* < 0.05) (Fig. 2-B).

### Rectus femoris

Significant main effects of surfaces [*F*_(2.21, 46.03)_ = 10.288, *p* < 0.001, $\:{\eta\:}_{p}^{2}$ = 0.329] and no significant main effects of phases [*F*_(1,21)_ = 4.240, *p* = 0.052], and no significant interaction between surfaces and phases [*F*_(3,63)_ = 2.237, *p* = 0.093] was observed. Pairwise comparisons showed that participants exhibited greater EMG activation on the stable surface and TRX conditions during the ascending phase than during the descending phase (*p* < 0.05). During the descent phase, greater EMG activation was observed on the Swiss ball than on other surfaces. In contrast, greater activation was observed at the Swiss ball compared to the BOSU during the ascent phase (*p* < 0.05) (Fig. 2-C).

### Vastus lateralis

Significant main effects of surfaces [*F*_(3,63)_ = 8.669, *p* < 0.001, $\:{\eta\:}_{p}^{2}$ = 0.292] indicated that the Swiss ball demonstrated higher VL EMG activity than the BOSU ball and Stable surface conditions (*p* < 0.05). Significant main effects of phases [*F*_(1,21)_ = 42.029, *p* < 0.001, $\:{\eta\:}_{p}^{2}$ = 0.667] were observed, indicating greater EMG activity in the VL muscle during the ascent phase compared to the descent phase (*p* < 0.05). No significant interaction between the surfaces and phases was observed [*F*_(1.93, 40.59)_ = 0.608; *p* = 0.544] (Fig. 2-D).

### Vastus medialis

Significant main effects of surfaces [*F*_(2.18, 45.72)_ = 4.587, *p* = 0.013, $\:{\eta\:}_{p}^{2}$ = 0.179] indicated that the Swiss ball demonstrated greater EMG activity than the BOSU ball and Stable surface conditions (*p* < 0.05). Significant main effects of phases [*F*_(1,21)_ = 20.094, *p* < 0.001, $\:{\eta\:}_{p}^{2}$ = 0.489] were observed, indicating greater EMG activity during the ascent phase than the descent phase (*p* < 0.05). No significant interaction between the surfaces and phases was observed [*F*_(3, 63)_ = 1.988, *p* = 0.125] (Fig. 2-E).

### Gluteus Maximus

Significant main effects of surfaces [*F*_(3,63)_ = 2.889, *p* = 0.042, $\:{\eta\:}_{p}^{2}$ = 0.121] indicated that the BOSU ball demonstrated higher GM EMG activity than the Stable surface condition (*p* < 0.05). Significant main effects of the phases [*F*_(1,21)_ = 87.197, *p* < 0.001, $\:{\eta\:}_{p}^{2}\:$= 0.806] indicated that greater EMG activity was produced during the ascent phase compared to the descent phase (*p* < 0.05). The significant interaction between surfaces and phases [*F*_(1.91, 40.13)_ = 3.509, *p* = 0.041, $\:{\eta\:}_{p}^{2}\:$= 0.143] were observed. Pairwise comparisons showed that the participants exhibited greater EMG activation in all conditions during the ascending phases than during the descending phases (*p* < 0.05) (Fig. 2-F).

## Discussion

This study aimed to evaluate and compare the muscle activation patterns of the lower extremity muscles during the ascent and descent phases of the BSS exercise performed on a stable surface and three different instability devices. The findings of this study demonstrated statistically significant differences in muscle activation across unstable conditions.

The primary finding of this study provided limited support for the hypothesis that during the ascending phase of BSS, muscle activation on all surfaces is greater than during the descending phase of BSS. The BF, VL, VM, and GM muscles were more highly activated during the ascent than during the descent phase on all surfaces. Similarly, the ST muscle on all unstable surfaces, the RF muscle on the stable surface, and the TRX showed greater muscle activation during the BSS ascent than during the descent. The findings of this study align with prior research on muscle engagement during exercises featuring similar movement patterns, including Rear Leg Elevated Split Squats and Split Squats^29^, as well as Monopodal Squats and Forward Lunges^43^. Some authors have indicated that the increased EMG activity during the ascent phase may be attributed to the slower muscle fiber conduction velocity during eccentric movements compared to that during concentric movements^44^. Eccentric muscle contractions generate greater force at lower levels of activation, thus controlling a descending movement (eccentric) with the same bodyweight elicits lower EMG activity than when ascending (concentric) with the same load^45–47^.

The second finding of this study was that the ST muscle exhibited greater activation on the TRX during the ascent phase than on the Swiss Ball. The greater ST activation observed in the TRX condition may be due to the suspension system inherently requiring more pronounced sagittal-plane balance control. In contrast, although the Swiss Ball allows multidirectional movement, its rolling tendency may place relatively greater demands on frontal plane stability. Given that the semitendinosus contributes primarily to anterior–posterior stabilization^48^, it may have been more actively recruited during TRX exercises than during Swiss Ball exercises. Similarly, the RF muscle showed greater activation on the Swiss Ball than BOSU during the ascent phase. The study’s findings indicated no significant differences in muscle activation during the ascending phase among the surfaces for the other muscles. During the descent phase, the ST muscle exhibited greater activation on the Swiss ball than on the BOSU ball. RF muscle showed greater activation on the Swiss Ball than on the other surfaces during the descent phase. In addition, the VL and VM muscles showed greater activation on the Swiss ball than on the stable surface and the BOSU during the descent phase. The study results showed no significant statistical differences in muscle activation during the descent phase among the surfaces for BF and GM muscles. The findings of this study align with and diverge from the existing literature on the influence of surface instability on lower limb muscle activation. Specifically, our results demonstrated that the RF, VL, and VM muscles exhibited greater activation when exercises were performed on a Swiss Ball, particularly during the descent phase. While previous studies have frequently reported a decrease in agonist muscle activation with increasing instability that attributed to the redistribution of motor control toward stabilizing musculature^25,49,50^, some investigations have also reported increased^18,20,51^ or unchanged activation levels under similar unstable conditions^24,26,52,53^. The results of the current study contrast with those reported by Saeterbakken and Fimland^23^, who observed reduced muscle activation during squat and BSS exercises performed on unstable surfaces. A key methodological difference lies in the application of instability: in their study, instability was introduced to the front leg using a foam pad, whereas in the present study, instability was applied to the rear leg using devices such as the BOSU, Swiss Ball, and TRX. Moreover, they implemented a 6-repetition maximum (6RM) external load, whereas the participants in our study performed the exercises using only their body weight. These differences in load intensity and instability configuration may significantly influence neuromuscular demands. Rear-foot instability, especially in the context of body weight, may place greater emphasis on postural control and proprioceptive engagement without overloading prime movers, potentially leading to higher muscle activation in specific phases of movement. The significant increase in RF activation during the descent phase on the Swiss Ball may be attributed to its biarticular anatomy and dual functional role. Although the RF is traditionally examined in relation to knee extension, it also serves as a primary hip flexor, particularly when postural demands are elevated under unstable conditions^54^. Performing the eccentric phase on an unstable surface increases the requirement for dynamic hip stabilization, which may enhance RF recruitment in the hip flexor. Prior studies have shown that RF contributes not only to joint movement but also to trunk and pelvic control during balance-challenging tasks^55^. Therefore, the elevated RF activity observed in this study may reflect its compensatory role in maintaining postural stability through hip flexion, rather than solely its function in knee extension^21,27,28^.

Another finding of the present study was the differential activation of major lower-limb muscle groups. Across all instability conditions and movement phases, the quadriceps muscles (RF, VL, VM) consistently exhibited higher EMG amplitudes than the hamstrings (BF, ST), whereas the GM showed intermediate activation levels. The relatively greater quadriceps involvement may reflect the BSS’s biomechanical emphasis on unilateral knee extension during both the ascending and descending phases^56^. Although the GM was actively engaged, particularly during the ascent phase, it did not reach the same activation magnitude as that of the quadriceps group. Furthermore, it should be noted that the BSS was performed in a vertical (up–down) manner, minimizing flexion of the rear leg, which may have limited the additional recruitment of the Gluteus Maximus and hamstring muscles. This could be due to the upright trunk posture maintained throughout the movement, which may reduce hip flexion and thereby limit the contribution of the GM^57^. Recent evidence further supports this explanation, indicating that maintaining a neutral (upright) trunk during the BSS significantly decreases activation of the Gluteus Maximus and Biceps Femoris compared to a forward-leaning trunk, likely due to altered joint angles and center of mass position^58^. Meanwhile, hamstring activity remained comparatively lower, likely due to the upright trunk posture described above, suggesting a stabilizing role rather than primary force generation^56^. These results suggest that the BSS, as performed in this study, is primarily a quadriceps-dominant exercise, with moderate gluteal and limited hamstring recruitment unless additional hip flexion or forward trunk lean is introduced into the exercise.

This study had several limitations that should be acknowledged. First, the sample consisted solely of trained male football (soccer) players, which limits the generalizability of the findings to other populations, such as female athletes, youth, and untrained individuals. Second, muscle activity was assessed using surface EMG only, which does not provide information on joint kinetics, kinematics or muscle-generated force output. Moreover, as with all surface EMG measurements, the data were susceptible to potential signal noise, crosstalk from adjacent muscles, and limitations in the detection of deeper muscle activity. Although electrode placement followed the SENIAM guidelines, these methodological constraints should be considered when interpreting the results. Third, all exercises were performed using body weight without external resistance, which may not fully reflect the typical strength-training conditions. Furthermore, the movement was performed with an upright torso and controlled tempo, potentially underestimating muscle activation in more dynamic or sports-specific trunk positions. Fatigue across repetitions was not monitored, and the study focused only on the acute responses. Additionally, proprioception was not assessed before the exercise. Future research should investigate loaded variations of the BSS and examine long-term neuromuscular adaptations.

### Practical applications

This study demonstrated that altering the rearfoot support surface during the BSS can selectively influence lower limb muscle activation, depending on the movement phase and targeted muscle group. For example, the Swiss Ball condition elicited the highest quadriceps activation, particularly during the eccentric phase. Generally based on the EMG-force relationship (linear and curvilinear relationships indicate that increased EMG is associated with higher force output)^59,60^, the higher levels of muscle activation would make it suitable for athletes aiming to enhance quadriceps strength and control under unstable conditions. The TRX suspension system was effective in increasing ST activation during the concentric phase, suggesting its use for hamstrings-focused training. Additionally, the BOSU surface promoted greater GM activity and may benefit programs targeting gluteal engagement and hip stability. These instability-based variations may have potential applications in strength or rehabilitation programs, particularly in contexts involving load restrictions or early stage recovery. However, the present study only measured muscle activity, and further research is required to confirm their effects on neuromuscular coordination, unilateral strength, or postural control.

## Data Availability

Data are available for research purposes from the corresponding author upon reasonable request. The individual de-identified participant data, statistical code, and additional materials supporting the findings of this study are available upon reasonable request from the corresponding author of this paper.
